# Synthesis of Non-Stoichiometric (TiNb)C_0.5_ with High Hardness and Fracture Toughness under HTHP

**DOI:** 10.3390/ma11071219

**Published:** 2018-07-16

**Authors:** Zhichao Zhang, Hu Tang, Yujiao Ke, Yu Li, Xiaochen Jiao, Changjian Geng, Yucheng Zhao, Mingzhi Wang

**Affiliations:** State Key Laboratory of Metastable Materials Science and Technology, Yanshan University, Qinhuangdao 066004, China; zhangzhichao@ysu.edu.cn (Z.Z.); tanghu_future@163.com (H.T.); Yujiaoke@126.com (Y.K.); A13933556327@163.com (Y.L.); m15249691052@163.com (X.J.); 13191820690@163.com (C.G.)

**Keywords:** mechanical alloying, non-stoichiometric compound, high temperature and high pressure, sintering, fracture toughness

## Abstract

Nonstoichiometric TiC_0.5_ and (TiNb)_0.5_ powders were prepared by the mechanical alloying process using Ti, Nb, and TiC powders as raw materials. Furthermore, the as-prepared TiC_0.5_ and (TiNb)_0.5_ powders were used as initial materials to fabricate TiC_0.5_ and (TiNb)_0.5_ compacts under high pressures and high temperatures (HTHP) of 5.5 GPa and 1200–1550 °C for 5 min. Phase identification and microstructure of the mechanical-alloyed powders and the sintered TiC_0.5_ and (TiNb)_0.5_ compacts were realized by an X-ray diffractometer and scanning electron microscope. The results indicate that the as-prepared TiC_0.5_ and (TiNb)_0.5_ powders have a similar crystal structure of face-centered cubic (FCC) to TiC. The sintered (TiNb)_0.5_ compact has good Vickers hardness (~16 GPa), and notably, excellent fracture toughness (~7.3 MPa·m^1/2^). The non-stoichiometric compound not only reduced the sintering temperature of covalent compounds, but also greatly enhanced the mechanical properties of the materials. Thus, we have provided a novel synthetic strategy for the production of a compound with high-strength covalent bonds.

## 1. Introduction

TiC and NbC have been highlighted for their excellent physical and chemical properties, such as high hardness, high corrosion resistance, good thermal stability and high thermal conductivity. These properties have allowed for their remarkable performance and provided them with high commercial value in the fields of metal cutting, milling, coating, and drilling [1,2,3,4,5,6]. However, these compounds are very difficult to fabricate by conventional sintering methods due to their high melting points and low self-diffusion coefficients. Consequently, the low sinterability and fracture toughness of these compounds have further limited their practical applications.

In the past few decades, extensive efforts have been made to reduce the sintering temperature and increase fracture toughness of these types of ceramics. Currently, the dominating method is to fabricate ceramic–metal composites, which are known as cermets or hardmetals. Generally, metal or alloy binders are introduced to improve the sinterability and fracture toughness of ceramics due to the deformation of plastics and crack bridging. Yue [7] fabricated TiC–TiB_2_-8 wt % Ni composite with hardness of 18.8 GPa and fracture toughness of 8.9 MPa·m^1/2^. Acharya [8] sintered TiC-10 wt % Ni–10 wt % NiB composite with hardness of 2759 HV and fracture toughness of 8.98 MPa·m^1/2^. Huang [9,10] sintered NbC-15 vol % 430 L cermet with a hardness of 13.6 GPa and fracture toughness of 7.3 MPa·m^1/2^ as well as NbC-24.5 wt % Co cermet with hardness of 11.8 GPa and fracture toughness of 7.6 MPa·m^1/2^. Nevertheless, the introduction of a binder will compromise the hardness and high-temperature application [11,12].

However, the use of a single-phased covalent bonding material with high combination properties of hardness and fracture toughness has been barely reported. Nano-twinned cubic boron nitride with hardness of 108 GPa and fracture toughness of 12.7 MPam^1/2^ sintered under the conditions of over 12 GPa and 1800 °C was reported [13] although this greatly exceeded the industrial conditions. Thus, a method for synthesizing pure ceramics with high hardness and fracture toughness under moderate conditions is urgently needed. The reduction of sintering conditions has been realized by introducing vacancies [14]. Xu reported that densified TiN compact with a relative density of 99.4% was fabricated at 5 GPa and 1200 °C for 10 min using the non-stoichiometric TiN_0.3_ powder, which allowed the sintering temperature to be significantly reduced [14]. Vacancy can reduce the strong covalent nature of covalent bonding compounds and promote atom diffusion during sintering process [15], which provides guidance for sintering refractory ceramic materials. However, sintered non-stoichiometric TiN_0.3_ bulk has similar low fracture toughness to stoichiometric TiN [14]. In this work, we prepared TiC_0.5_ and (TiNb)C_0.5_ powders by mechanical alloying (MA) and used them as starting powders to synthesize single-phased TiC_0.5_ and (TiNb)C_0.5_ compacts at relatively low temperatures without binders. With the addition of Nb element, the single-phased (TiNb)C_0.5_ compact was successfully fabricated with high hardness (~16 GPa) and fracture toughness (~7.3 MPa·m^1/2^). Our findings have provided a universal synthetic route for refractory ceramics with high hardness and fracture toughness.

## 2. Materials and Methods

TiC_0.5_ and (TiNb)C_0.5_ powders were prepared through mechanical alloying (MA) with Ti powder (purity >99.5%, ~30 μm), Nb powder (purity >99.5%, ~37 μm) and TiC powder (Purity >99.5%, 1–10 μm). The starting materials were purchased from Qinhuangdao ENO High-Tech Material Development Co., Ltd. The powder mixture was sealed in a tungsten carbide (WC) jar (volume: 500 mL) with WC balls in an argon atmosphere. The ball-to-powder weight ratio was 20:1. MA was conducted in a vario-planetary mill (Fritsch, Markt Einersheim, Germany) at a rotation speed of 700 rpm for 18 h. After this, the powders were treated in a vacuum oven at 600–800 °C for 2 h. The as-prepared powders were sintered under high temperatures and high pressures (HTHP) in a hexahedron anvils press (CS-1B, Guilin Guiye Heavy Industries Co., Ltd., Guilin, China). The final consolidated compacts were ground by using a diamond grinding wheel to remove the graphite layer, before the compacts were polished with diamond paste to 0.1 μm.

X-ray diffraction (XRD; D/max-2500PC, Rigaku, Tokyo, Japan) with Cu Kα_1_ radiation (λ = 0.15406 nm, 40 kV, 200 mA) was employed for phase identification. Rietveld refinements of the XRD patterns were carried out by GSAS program [16]. The average grain size *D* and strain of grains ε were roughly calculated from the XRD data by using the Williamson–Hall method [17,18,19] according to the following equation:

*B*cos*θ* = (*Kλ*/*D*) + (4εsin*θ*) (1)

where *λ* is the wavelength of Cu Kα_1_; *B* is the full width at half maximum; and *K* is 0.89.

Fractured surface morphology was observed by scanning electron microscopy (FE-SEM; S-4800II, Hitachi, Tokyo, Japan). Vickers hardness was measured using a Vickers diamond indenter (FM700, Future-tech., Kanagawa, Japan) with a load of 500 g and a dwelling time of 10 s. Fracture toughness (*K*ic) was measured by the indentation method under an indentation load of 10 kg using the following expression that was derived by Shetty et al. [20]:

(2) $$\;K\mathrm{ic}\;=\;0.0889{\left(\frac{HvP}{4l}\right)}^{1/2}\;$$

where *H*v is the Vicker’s hardness; *P* is the indentation load; and *l* is the crack length. The elastic modulus was determined by the ultrasonic method.

Based on the first principles density functional theory, the CASTEP code of materials studio software (5.0, Accelrys, San Diego, CA, USA) was adopted for XRD simulations of the stoichiometric TiC, NbC, and Ti_2_C powder.

## 3. Results

**Figure 1:**
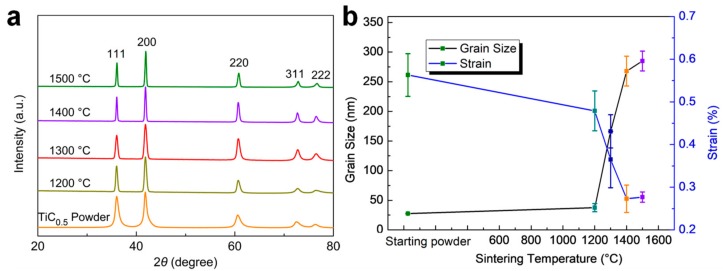
(**a**) X-ray diffraction (XRD) patterns of as-prepared non-stoichiometric TiC_0.5_ powder and TiC_0.5_ compacts sintered at different temperatures; and (**b**) grain size and strain of as-prepared non-stoichiometric TiC_0.5_ powder and compacts sintered at 5.5 GPa and different temperatures.

Diffraction peaks of the as-prepared non-stoichiometric TiC_0.5_ powder are consistent with that of crystal TiC. The Ti phase was not detected as shown in the XRD pattern in Figure 1, indicating that non-stoichiometric TiC_0.5_ powders were successfully synthesized by MA. TiC_0.5_ compacts that were sintered at HTHP showed no phase transition (Figure 1a). The full width at half maximum (FWHM) of the diffraction peaks shows a notable change as the sintering temperature increases, which indicates the drastically increased grain size. The grain size of the sintered compact is basically the same as that of the as-prepared powder when the sintering temperature is below 1200 °C. Furthermore, it increases rapidly as the sintering temperature increases above 1200 °C. On the contrary, the strain of grains becomes decreased with the increasing temperature. The rapid grain growth and decrease in intergranular strain suggest that the sintering behavior is more prominent above 1200 °C and the stress release caused by MA facilitated the elimination of an intergranular strain.

**Figure 2:**
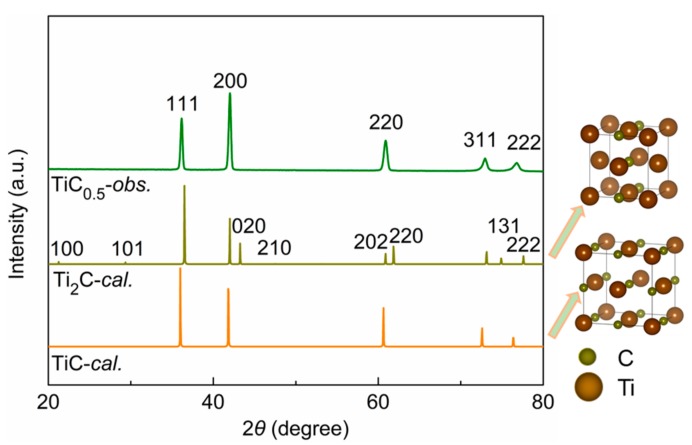
XRD patterns of theoretically calculated and experimentally obtained TiC_0.5_.

The Ti and C atomic ratio of 1:0.4982 (shown in Table 1) was accurately measured by spectrophotometry. The XRD results indicate that the obtained TiC_0.5_ have a similar crystal structure of face-centered cubic (FCC) to TiC (Figure 2 and Table 1). Halving the amount of carbon vacancies does not lead to the formation of Ti_2_C with a tetragonal structure. Therefore, the crystal structure of TiC_0.5_-*obs* shows a distinctive difference to that of the Ti_2_C, which have an ordered arrangement of C atoms in the crystal structure. It can be inferred that the as-prepared non-stoichiometric TiC_0.5_ powder prepared in this work still has a face-centered cubic (FCC) structure although the distribution of C atoms is disordered [21,22]. It is possible that due to C concentration gradient and MA, the C atoms diffuse into the crystal lattice of Ti during the process of MA. As long as the C atom in Ti crystal lattice exceeds its saturation, the non-stoichiometric TiC_0.5_ based on the TiC lattice structure can be formed.

**Figure 3:**
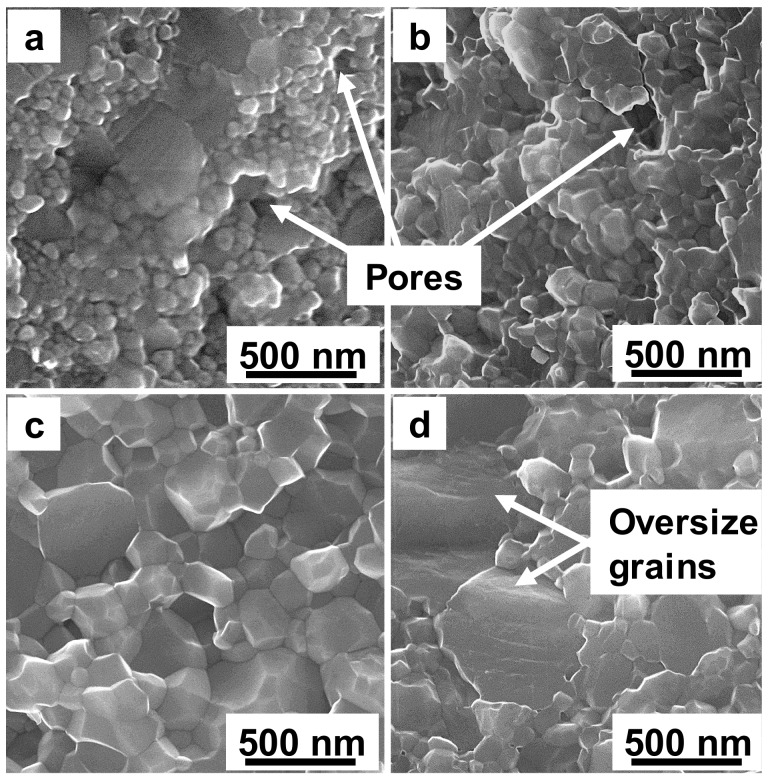
Fractured surface of the TiC_0.5_ compacts sintered at high temperature and high pressure (HTHP): (**a**) 1200 °C; (**b**) 1300 °C; (**c**) 1400 °C; and (**d**) 1500 °C.

The fractured surface of the TiC_0.5_ compacts that were sintered at HTHP exhibits a densification process, which is coupled with the grain growth and grain boundary formation as the sintering temperature increases (Figure 3). At 1400 °C, homogeneous grain size distribution is observed. In contrast, at 1500 °C, a small number of grains tend to grow oversized in the sintered compact, which is the typical phenomenon of abnormal grain growth and overheat. The fractured surface indicates that the grain size increases as the sintering temperature increases. The grain size is up to 200–300 nm when the temperature is above 1400 °C, which is consistent with the XRD data (as shown in Figure 1b).

**Figure 4:**
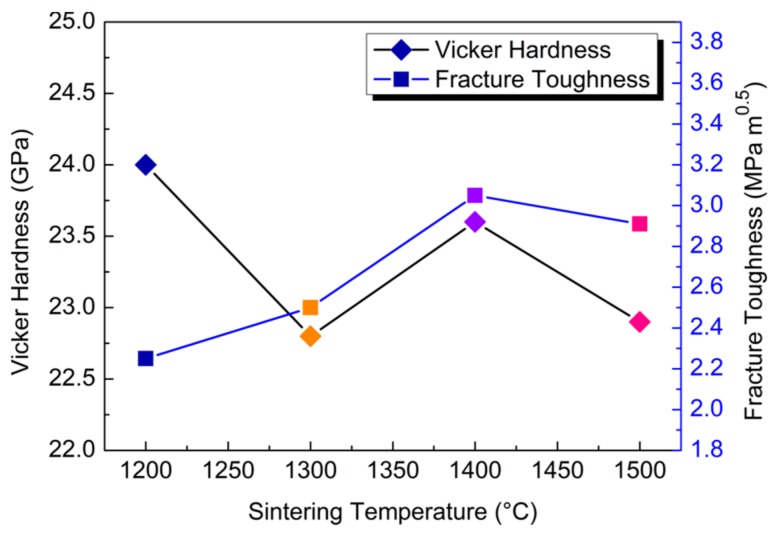
Microhardness and fracture toughness of TiC_0.5_ compacts sintered at HTHP.

The curves of microhardness and fracture toughness of TiC_0.5_ compacts sintered at HTHP are shown in Figure 4. The hardness values are in the range of 22–24 GPa, which increases with an increase in the sintering temperature. The microhardness value of the non-stoichiometric TiC_0.5_ is lower than the intrinsic microhardness of normal TiC (27 GPa) [6], which may be due to the C vacancies. The density of Ti–C bonds is lower than that of the stoichiometric TiC, which further leads to the lower microhardness value of the non-stoichiometric TiC_0.5_.

The fracture toughness of the compacts increases gradually as the sintering temperature increases (Figure 4), which reaches the peak value of 3.05 MPa·m^1/2^ at 1400 °C. After this, it decreases as the sintering temperature continues to rise. In this case, the fine grains have more interfaces, with the complete interface having enhanced their interfacial strength and further increased their toughness [23,24].

**Figure 5:**
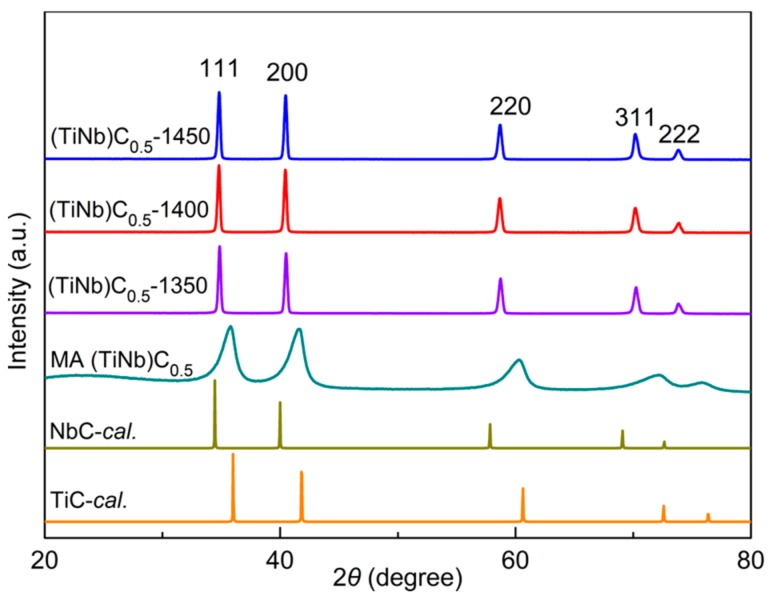
XRD patterns of as-prepared non-stoichiometric (TiNb)C_0.5_ powder, (TiNb)C_0.5_ compacts sintered at different temperatures and theoretically calculated TiC and NbC.

Although the non-stoichiometric TiC_0.5_ can be synthesized at lower temperatures, the mechanical properties can still be improved, especially the fracture toughness. In order to enhance the mechanical properties, we chose the transition metal Nb to substitute Ti in the non-stoichiometric TiC_0.5_ lattice. The atomic radius of Nb is 1.48 Å, which is similar to that of Ti (1.45 Å). Furthermore, the NbC compound has same crystal structure as TiC. Theoretically, the Nb can substitute Ti at any ratio. Here, for obtaining non-stoichiometric (TiNb)_0.5_, the equimolar powders of Nb and TiC were well-mixed by MA. XRD results show that the diffraction peaks of Nb completely disappeared (Figure 5). The diffraction peaks are consistent with that of TiC-*cal* peak positions. It is possible that the crystal structure of TiC may act as the basic structure of (TiNb)C_0.5_. During MA process, C atoms diffused into Nb lattice due to the concentration gradient and MA.

However, the synthesized (NbTi)C_0.5_ compacts maintain the original crystal structure of FCC after sintering at HTHP (1350–1550 °C, 5.5 GPa), which is shown in Figure 5. The lattice parameters (4.4403 Å) of (TiNb)_0.5_-*obs* is between that of TiC-*cal* (4.4403 Å) and NbC-*cal* (4.5019 Å) (Table 1), which depends on the atomic radius (Ti and Nb) in the lattice site.

**Figure 6:**
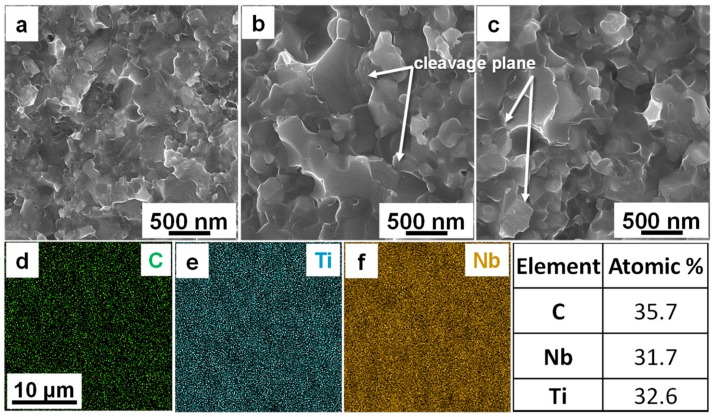
Fractured surface of (TiNb)C_0.5_ compacts sintered at different temperatures and element surface distribution of samples. (**a**–**c**), Microscopic morphology of the fracture of (TiNb)C_0.5_ samples sintered at 1350 °C (**a**), 1450 °C (**b**), and 1550 °C (**c**), respectively. (**d**–**f**), Carbon atom (**d**), Titanium atom (**e**), Niobium atom, and (**f**) surface distribution of samples after being sintered at 1550 °C.

The pressure largely affects the density and suppresses grain coarsening of the sintered compact during HTHP by controlling the atom diffusion, while the temperature acts on increasing grain size by promoting atom diffusion [25,26,27]. At 1350 °C, the edges and corners of the fine particles of the sintered compacts are misty and were loosely connected as shown in SEM photographs of the fracture surface (Figure 6a). When the temperature reaches 1450 °C, the dense morphology indicates that the compacts were well sintered with dramatic grain growth. Figure 6d–f show the mapping analysis of the elements of C, Ti, and Nb which are all uniformly distributed in the sintered compact with an atomic ratio of approximately 1:1:1, suggesting that the sintered compacts consist of single-phased non-stoichiometric (TiNb)_0.5_.

**Figure 7:**
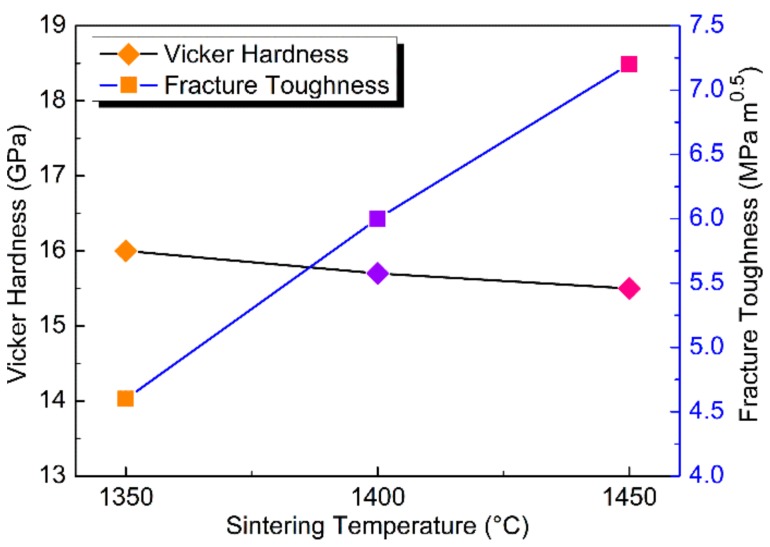
Microhardness and fracture toughness of compacts sintered at HTHP.

For polycrystalline materials, the mechanical properties rely substantially on the microstructures. Figure 7 shows the microhardness and fracture toughness of the sintered compacts at HTHP. The hardness of (TiNb)C_0.5_ compacts are almost constant as the sintering temperatures increase. However, because of the addition of Nb, the fracture toughness of the (TiNb)C_0.5_ compacts are remarkably improved. The compact synthesized at a higher temperature of 1550 °C has the optimum fracture toughness of 7.3 MPa·m^1/2^, which is twice of that of TiC_0.5_, as shown in Figure 7.

As we can see in Figure 7, the fracture toughness of (TiNb)C_0.5_ samples increase as the sintering temperature rises, which can be reasonably explained by the bonding situation of grains. Well-sintered compact can be obtained in higher temperatures as this increases the energy of atomic diffusion. From SEM photographs (Figure 6), we found that at 1450 and 1550 °C the samples display obvious cleavage surface. Under external stress, the crack would propagate along the cleavage surface. However, the compacts were well sintered with strong grain boundaries and rapid densification. The formed strong boundaries can control the propagation of crack and compel the crack to pass into the grains. The propagation path of the crack was largely shortened, which leads to an increase in the fracture toughness. The synthesized non-stoichiometric (TiNb)C_0.5_ compacts have satisfactory mechanical properties of high hardness of ~16 GPa and excellent fracture toughness of ~7.3 MPa·m^1/2^. In fact, other studies have reported that refractory ceramics (transition metal carbide or nitride) with high hardness and fracture toughness generally can be obtained by adding a metal binder with a low melting point [7,8,28]. However, the great differences of physical and chemical properties between the ceramics and added metals lead to the deterioration of stability.

## 4. Conclusions

In summary, we synthesized single-phase non-stoichiometric compounds of TiC_0.5_ by the MA and HTHP processes. The synthesized TiC_0.5_ has the same crystal structure of FCC to stoichiometric TiC. The hardness values and fracture toughness of sintered TiC_0.5_ are ~22–24 GPa and ~2.2–3.0 MPa·m^1/2^, respectively. By adding equimolar Nb, (TiNb)C_0.5_ with FCC structure was obtained, having mechanical properties of hardness of ~16 GPa and fracture toughness of ~7.3 MPa·m^1/2^. Noticeably, the fracture toughness was drastically improved. Based on this result, we will focus on synthesizing multi-elemental refractory carbides or nitrides with high hardness of fracture toughness in the next study.

## Figures and Tables

**Table 1:** Space group and lattice parameters of the experimental matter.

Compound	Space Group	Lattice Constant (Å)		
		A	b	c
TiC-*cal*.	FM-3M	4.3161	4.3161	4.3161
Ti_2_C-*cal*.	P4/MMM	4.2997	4.1792	4.2997
TiC_0.5_-*obs*. (TiC_0.4982_)	FM-3M	4.3193	4.3193	4.3193
NbC-*cal*.	FM-3M	4.5019	4.5019	4.5019
(TiNb)C_0.5_-*obs*.	FM-3M	4.4403	4.4403	4.4403

Notes: Experimental lattice constants were calculated from XRD patterns of TiC0.5 and (TiNb)C0.5.
